# Early sepsis recognition: how difficult can this be?

**DOI:** 10.1186/s13054-024-04975-3

**Published:** 2024-06-05

**Authors:** Maria-Evangelia Adami, Jesper Eugen-Olsen, Evangelos J. Giamarellos-Bourboulis

**Affiliations:** 1 Fourth Department of Internal Medicine, Medical School, ATTIKON University General Hospital, National and Kapodistrian University of Athens, 1 Rimini Str, 124 62 Athens, Greece; 2 Department of Clinical Research, Copenhagen University Hospital Amager and Hvidovre, Hvidovre, Denmark; 3 Hellenic Institute for the Study of Sepsis, Athens, Greece

We read with interest the letter of Paliwal et al. [1] addressing four specific concerns for the results of our SUPERIOR trial. In this reply, we need to clarify that SUPERIOR was designed to address an existing unmet need. The qSOFA score is introduced with the Sepsis-3 definitions to assist early recognition when patients present in the Emergency Department (ED). It consists of 3 clinical variables: tachypnea, mental confusion and hypotension [2]. When two or more signs are found, early management of sepsis should start. However, several patients with suspicion of infection are triaged at the ED and they meet only one sign of qSOFA. How can the presence of one sign of qSOFA be interpreted in the suspicion of an infection?

In our SUPERIOR trial, we consider the use of soluble urokinase plasminogen activator receptor (suPAR) as an enrichment tool to select among patients with one qSOFA [3]. The first concern by Paliwal et al. [1] is the time of sampling for cultures. Sampling for microbiology was done before intervention. This is indicated by our pathogen isolation rate being almost 30% as it is the average of pathogen identification in sepsis. Their second concern is the impact of the use of SIRS criteria in the retrospective cohort but not in the SUPERIOR trial. As explicitly stated [3], to tackle any confounding coming from SIRS, it was designed to follow-up for 28-days all patients screened for the SUPERIOR trial with one sign of qSOFA. Those enrolled in the trial with suPAR ≥ 12 ng/ml and were treated with placebo and those with suPAR less than 12 ng/ml differed significantly in 28-day mortality (17.0% vs 6.6%). This confirmed that suPAR could indicate the risk of death among patients independently than SIRS.

One third concern by Paliwal et al. [1] is the impact of the lack of study power on the interpretation of the findings. The significance of the early meropenem intervention is confirmed by our logistic regression model and this limits the uncertainty coming from the inability to enroll a fully powered cohort. Their fourth concern is ethical issues coming from the use of placebo medication. SUPERIOR is designed for a patient population with suspicion of infection where no guidance for early intervention exists. As such, no ethical issues are raised. Meropenem was selected as an intervention due to the broad-spectrum coverage including both pathogens producing extended-spectrum β-lactamases and anaerobes making the drug appropriate for a broad range of infections in settings with high levels of antimicrobial resistance. However, the essence of the intervention is not for the specific drug but for early start of antibiotics which may be selected by local antimicrobial surveillance teams.

The SUPERIOR trial frames a patient population without defined sepsis but at risk of progression into organ dysfunction. No guidance exists for these patients and results of SUPERIOR introduce the need of large-scale RCTs to verify findings, as Paliwal et al. agreed [1].

## Data Availability

No new data are produced by this letter of correspondence.

## Abbreviations

ED: Emergency department
qSOFA: Quick SOFA
RCT: Randomized controlled trial
SIRS: Systemic inflammatory response syndrome
SOFA: Sequential organ failure assessment
suPAR: Soluble urokinase plasminogen activator receptor

## References

[1] Paliwal B, Kothari N, Saha T (2024). qSofa combined with suPAR for early risk detection and guidance of antibiotic treatment in emergency department: a bit sweet and a bit sour randomized controlled trial. Crit Care.

[2] Singer M, Deutschman CS, Seymour CW (2016). The third international consensus definitions for sepsis and septic shock (Sepsis-3). JAMA.

[3] Adami ME, Kotsaki A, Antonakos N (2024). qSOFA combined with suPAR for early risk detection and guidance of antibiotic treatment in the emergency department: a randomized controlled trial. Crit Care.

